# Proteomics profiling reveals mitochondrial damage in the thalamus in a mouse model of chronic migraine

**DOI:** 10.1186/s10194-023-01646-6

**Published:** 2023-09-05

**Authors:** Wei Xie, Ruibing Li, Wenjing Tang, Zhenjie Ma, Shuai Miao, Chenhao Li, Chunxiao Yang, Bozhi Li, Tao Wang, Zihua Gong, Yue Zhou, Shengyuan Yu

**Affiliations:** 1 Department of Neurology, the First Medical Center, Chinese PLA General Hospital, Beijing, China; 2 Medical School of Chinese PLA, Beijing, China; 3 Department of Laboratory Medicine, the First Medical Center, Chinese PLA General Hospital, Beijing, China; 4 School of Medicine, Nankai University, Tianjin, China; 5 College of Life Science, Northwest University, Xi’an, Shanxi China

**Keywords:** Chronic migraine, Thalamus, Mitochondrial damage, Proteomics

## Abstract

**Background:**

Migraine, a complex brain disorder, is regarded as a possible clinical manifestation of brain energy dysfunction. The trigeminovascular system is considered the basis for the pathogenesis of migraine, hence we depicted the proteomics profiling of key regions in this system, then focusing on protein alterations related to mitochondrial function. The aim of this study is to illustrate the role of mitochondria in migraine.

**Methods:**

A mouse model of chronic migraine (CM) was established by repeated nitroglycerin (NTG) stimulation and evaluated by von-Frey filaments, a hot plate and a light-dark box. Differentially expressed proteins (DEPs) in some subcortical brain regions of the trigeminovascular system were screened through liquid chromatography-tandem mass spectrometry (LC‒MS/MS) to analyse the specificity of key signaling pathways in different brain regions. And then mitochondrial function, structure and dynamics were determined by qPCR, ELISA, and transmission electron microscope (TEM). Finally, the effect of mitochondrial intervention-Urolithin A (UA) on CM was investigated.

**Results:**

Repeated NTG injection triggered photophobia, periorbital and hind paw allodynia in mice. The proteomics profiling of CM model showed that 529, 109, 163, 152 and 419 DEPs were identified in the thalamus, hypothalamus, periaqueductal grey (PAG), trigeminal ganglion (TG) and trigeminocervical complex (TCC), respectively. The most significant changes in the brain region-specific pathways pointed to thalamic mitochondrial impairment. NTG induced mitochondrial structural disruption, dysfunction and homeostatic dysregulation, which could be partially attenuated by UA intervention.

**Conclusion:**

Our findings highlight the involvement of mitochondrial damage in the thalamus in central sensitization of CM, which provides evidence of possible metabolic mechanisms in migraine pathophysiology.

## Background

 Migraine is a common, recurrent, hereditary neurovascular headache disorder, which is the second leading cause of years lived with disability in the world according to the Global Burden of Diseases (GBD) [1]. The current best estimate of global migraine prevalence is approximately 14–15% [2]. Chronic migraine (CM) usually transforms from episodic migraine (EM), affecting 1–2% of the general population. It is characterized by high disability, poor treatment response, frequent recurrences, a high ratio of medication-overuse, and more neuropsychiatric comorbidities [3]. Despite the enormous clinical impact, the pathophysiological mechanisms underlying the development and chronicity of migraine remain poorly understood.

In addition to the classical theory of the trigeminovascular system [4], impairment in mitochondria has been proposed as a potential contributor to the pathophysiology and susceptibility of migraine [5]. Studies have found that the prevalence of migraine in patients with mitochondrial disease is more than twice that in the general population [6]. Migraine attacks are more severe and last longer in patients with mitochondrial encephalopathy with lactic acidosis and stroke-like episodes (MELAS) [7], which is a single-gene inherited disease of mitochondrial DNA (mtDNA), suggesting an association between mitochondrial deficiency and migraine susceptibility and severity. In our previous study, we found that cortical spreading depression (CSD) could aggravate cerebral mitochondria injury under hypoxic conditions based on a rat CSD model, whereas flunarizine can alleviate brain mitochondrial damage under both normoxic and hypoxic conditions [8]. However, the association between mitochondria and CM remains not clear. Deciphering their causal relationship would be a challenge in revealing new mechanism of CM pathogenesis.

As an increasingly powerful tool, liquid chromatography-tandem mass spectrometry (LC‒MS/MS) analysis has been applied to unmask the disease pathophysiology. This could make it possible to identify and quantify a few thousand proteins from a limited amount of sample in a single experiment and screen differentially expressed proteins (DEPs) in a complex mixture, thus providing new hints about disease pathogenesis [9]. In previous studies on migraine and other neurological disorders, it had been performed to screen out key factors, significant pathways, and explore potential biomarkers. Adriana et al. [10] explored the analgesic effects and mechanism of endocannabinoid enhancement in rat and human meningeal tissues by activity-based protein profiling and LC‒MS/MS. Lipids are not only a major form of energy storage but also a source of inflammation and pain signaling molecules. Using LC‒MS/MS, Castor K et al. [11] found that plasma and cerebrospinal fluid (CSF) lipid metabolism are associated with CM pathology. In 2022, a plasma transcriptomic and metabolomic study in patients with migraine during and between headache attacks showed that one of the significantly different pathways between migraine patients and healthy individuals was associated with mitochondrial dysfunction [12].

Currently, most studies emphasized the plasma or serum of humans, as well as the cortex of humans and rodents [13–17], while less attention has been given to subcortical brain regions. Therefore, we performed quantitative proteomics analysis in five important subcortical brain regions of the trigeminovascular system to reveal the crucial alteration in key brain region and decipher the specificity of key signaling pathways in different brain regions. The emphasis was placed on the changes in proteins related to mitochondrial function in each brain region.

## Materials and methods

### Animals

All male C57BL/6J mice (21–32 g) were obtained from SiPeiFu Biotechnology Co., Ltd. (Beijing, China). Mice were housed in a facility with a controlled temperature of 23 ± 2 ℃, humidity of 50 ± 10%, a 12 h light-dark cycle, and free access to food and water. Mice were left to acclimatize for 1 week prior to starting experiments. The animals were assigned to the experimental group using a randomized program (http://www.randomizer.org/). The sample size was calculated by G*Power (ver. 3.1.9.7) based on the repeated measures design (α = 0.05, power = 0.8, effect size = 0.32, between 0.25 and 0.4) and the results showed the total sample size was 18, that means 9 mice per group [18]. All experimental procedures were approved by the Ethics Committee of the Chinese PLA General Hospital (ethics no. S2022-536-01), and followed the ethical guidelines of the International Association for the Study of Pain in conscious animals [19].

### NTG-induced CM mouse model

**Fig. 1:**
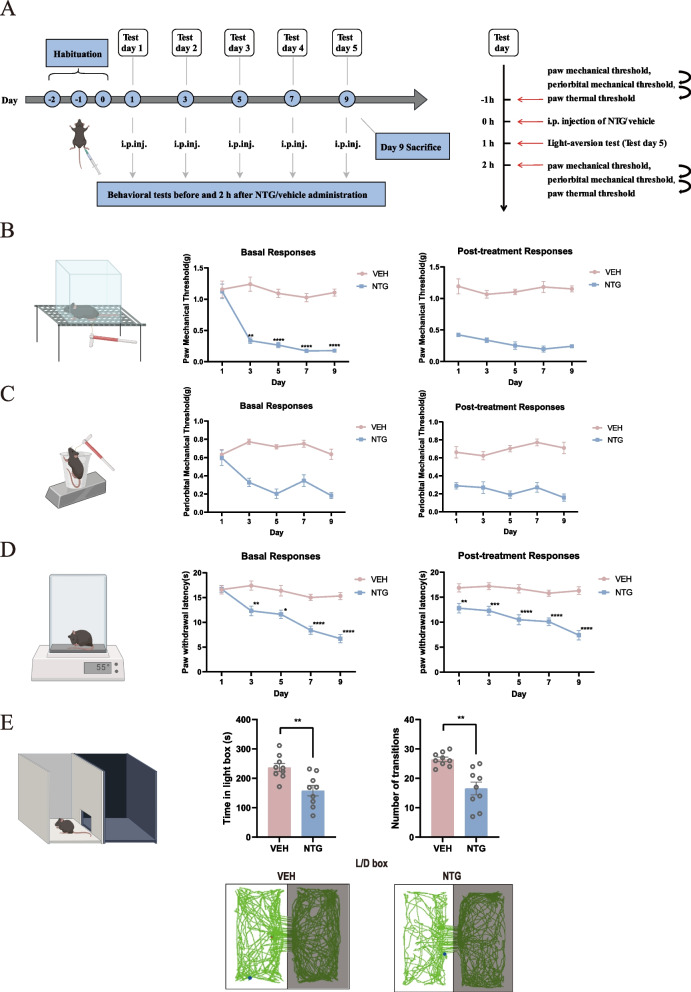
Experimental process of modeling and behavioral testing. **A** Flow chart of the experiment. **B-D** The basal and post-treatment mechanical pain thresholds of the hind paw (**B**), periorbital area (**C**) and the thermal pain threshold of the hind paw (**D**) during the injection of NTG. The data are presented as the mean ± SEM; two-way ANOVA with the Bonferroni post hoc test. **E** The time in light box and the number of transitions for each group during a total of 10 min. The data are presented as the mean ± SEM. Independent-sample t test. (*n* = 9 per group, *p < 0.05, **p < 0.01, ***p < 0.001, ****p < 0.0001)

Referring to previous studies, we applied a validated mouse model of CM, which was established by repeated intraperitoneal (i.p.) injection of nitroglycerin (NTG) (10 mg/kg) every second day for a total of 5 injections to closely mimic the recurrent attacks of CM in humans (Fig. 1A) [20–22]. NTG (Beijing Yi Ming, Beijing, China) was prepared from a stock solution of 5.0 mg/ml nitroglycerin in 30% alcohol, 30% propylene glycol, and 0.9% saline. The vehicle control contained 0.9% saline, 6% propylene glycol, and 6% alcohol and the method of administration was the same as that of NTG. Behavioral tests were carried out prior to the mice being sacrificed (Fig. 1).

### Drug administration

To investigate the role of mitochondria in CM, the mitochondria protective drug, Urolithin A (UA, MedChemExpress/MCE, America) was administered to the mice. As a natural gut microbiome-derived metabolite, UA displayed potent anti-inflammatory, anti-oxidative and anti-ageing properties [23–25]. UA (50 mg/kg) was intraperitoneally injected once a day for 9 consecutive days before NTG or vehicle treatment and after baseline threshold measurement (Fig. 8A). UA was dissolved in 10% DMSO, 40% PEG300, and 5% Tween 80. An equivalent volume of solvent corresponding to UA was used as a vehicle control. Mice were randomly divided into 4 groups: (1) NTG + UA, (2) NTG + VEH, (3) SHAM + UA, (4) SHAM + VEH.

### Behavioral tests

To obtain accurate and objective results, the mice were habituated in the behavior room for several days prior to testing to reduce stress. The behavior room was quiet and hygienic, with moderate light, good ventilation and no smell. Behavior experiments were carried out at a fixed time every day (between 9:00 am and 3:00 pm). All the behavioral tests were performed in the same batch of mice. Eventually, six mice with significant changes in behavioral testing in each group were selected for further proteomics analysis. A fixed investigator was responsible for behavioral assessments and was blinded to the treatment during behavioral measurements.

#### Mechanical withdrawal threshold test

The mechanical withdrawal threshold was evaluated using von Frey filaments (range from 0.008 to 2 g). The periorbital and hind paw mechanical thresholds of the mice were assessed before and 2 h after i.p. injection of NTG or vehicle on each test day. Based on the traditional up-down method, a novel statistical algorithm that calculates the exact thresholds was used. Briefly, the middle weight 0.16 g was first applied. The stimulus would increase one weight if there is a negative response (O) and decrease one weight with a positive response (X). The interval between two stimuli was more than 30 s. Following the breaking point (OX/XO), four other stimuli were applied resulting in a certain pattern of responses. The 50% threshold can be obtained by uploading the weight of the final filament and XO response pattern using the online tool [26].

For the hind paw assessment, each mouse was placed separately in transparent plastic chambers (8 × 15 cm) which were positioned on a wire mesh grid. The mice were acclimated in the chamber for 30 min per day at least 3 days before testing. The von Frey filaments were perpendicularly applied to the plantar surface of the hind paw. Brisk paw withdrawal, flinching, lifting, or licking during stimulation or immediately after the removal of the filament were described as positive responses. For the periorbital assessment, each mouse was tested in a 4 oz paper cup that was placed in the plexiglass box. The mice were habituated in the cups for 30 min during each of the 3 days before measurements. The periorbital region was tested, including the caudal region of the eye to approximately the midline. A positive response included the mouse vigorously stroked its face with the ipsilateral fore paw, head shaking or retraction of head from the stimulus.

#### Thermal withdrawal latency

The hind paw thermal thresholds were assessed before and 2 h after NTG or vehicle administration on each test day using a hot plate. After habituation, the mice were individually placed on the surface of a hot plate (Bioseb, France) with a preset constant temperature of 55 ℃. The response latency in seconds to the latency of hind paw withdrawal, licking, or jumping was recorded. To avoid tissue damage, the maximum response time was fixed at 20 Sect. [27]. Each mouse was tested three times with an interval of 5 min, and the average value was taken as the thermal withdrawal latency.

#### Light-aversive test

A light-dark box was used to assess light-aversive behaviors of the mice 1 h after NTG or vehicle injection on the last test day. The light-dark box (Xinruan Information Technology Co. Ltd, Shanghai, China) consisted of two equal-size zones: a light chamber and a dark chamber (20 cm × 15 cm × 30 cm), which were separated by a dark insert (Med Associates) with an open channel (6 cm × 6 cm) to allow the mice to explore the two chambers freely. After injection of NTG or vehicle, mice were individually placed in the middle of the light chamber (inside = 1000 lx) and allowed to explore both chambers for 10 min. The trajectory of movement was video-recorded and analysed using Supermaze software. The total distance of motion, the transition between two chambers, and the time spent in each chamber were calculated.

### Label-free LC‒MS/MS-based proteomics analysis

**Fig. 2:**
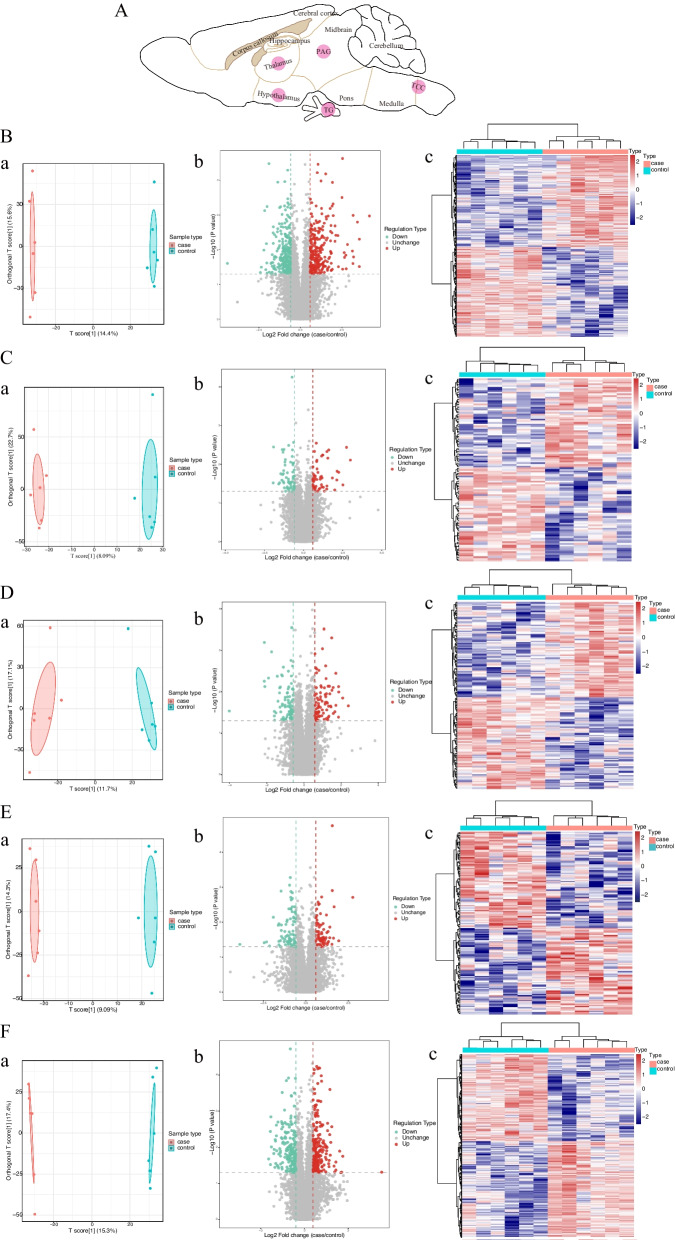
Differential expression of proteins in five brain regions. **A** Schematic diagram of five key subcortical brain regions of the trigeminovascular system, marked by pink circles. **B**-**F** The OPLS-DA (**a**), volcano plots (**b**) and clustering heat maps (**c**) analysis of proteins in the thalamus (**B**), hypothalamus (**C**), PAG (**D**), TG (**E**) and TCC (**F**). **a** In the OPLS-DA model, the proteins of five brain regions were significantly separated between the NTG and VEH groups. **b** and **c** Volcano plots and clustering heat maps illustrated the relative expression levels of DEPs in two groups, with red representing up-regulated proteins and green/blue representing down-regulated proteins (*n* = 6 in each group)

On test day 5, the mice were sacrificed 12 h after the last NTG or vehicle injection. The mice were anesthetized by intraperitoneal injection of sodium pentobarbital (50 mg/kg), and the brain tissues (thalamus, hypothalamus, PAG, TG and TCC) were harvested immediately according to Paxinos and Franklin’s Mouse Brain in Stereotaxic Coordinates (second edition) and stored at -80 ℃ (Fig. 2A). Tissue lysates were homogenized and digested following the filer-aided sample preparation (FASP) method with 10 kDa MW Amicon Ultra 0.5 centrifugal filters (Merck Millipore, Germany) [28]. Protein samples (100 µg) were dissolved in 8 M urea, reduced with dithiothreitol (Sigma, USA) at a final concentration of 10 mM at 37 ℃ for 30 min and then alkylated with 50 mM iodoacetamide (Sigma, USA) for 30 min in the dark at room temperature. Subsequently, protein solutions were filtered with 50 mM ammonium bicarbonate for 15 min at 10,000 g. Then, proteins were digested with lys C and trypsin (Promega, USA) mixture (proteins: enzyme (mass ratio) = 50 : 1 overnight at 37 ℃. Peptides were desalted with a SOLA HRP cartridge (Thermo Fisher, USA) according to the manufacturer’s procedure. Briefly, peptides were loaded onto the cartridge, washed with 0.1% trifluoroacetic acid (TFA), and eluted with 60% I in 0.1% TFA. Finally, peptides were reconstituted with 100 µL 0.1% formic acid (FA).

The prepared peptides were analysed by liquid chromatography (Ultimate RSLC nano 3000, Thermo Fisher, USA)-tandem mass spectrometry (Q-Exactive HF-X, Thermo Fisher). Two micrograms peptides were separated with an analytical column (Acclaim PepMap C18, 2 μm, 100 A, 75 μm x 25 cm) in 150 min. Mobile phase: A: 0.1% formic acid in water; B: 0.1% formic acid in 80% acetonitrile; gradient: 1 − 8% B in 4 min, 8 − 30% B in 136 min, 30 − 90% B in 6 min, 100% − 100% B in 4 min; flow rate: 400 nL/min. Mass spectrometer: spray voltage: 2.1 kV; capillary temperature: 300 ℃; S-lens: 50%; collision energy: 32% HCD; resolution setting: first level 60,000@m/z 200, two Level 30,000@m/z 200; Max IT: full MS 20 ms, full MS/MS 45 ms; parent ion scanning range: m/z 400–1040; product ion scanning range: start from m/z 200; number of windows: 80; Isolation window: 8 Th. One blank after each sample was run to prevent sample carryover.

### Bioinformatics analysis

Data were imported into DIANN V 1.8.0, and targeted extracted with a predicted mouse proteomics database (Uniprot_Mus_musculus_202102, 55,470 sequences) in library-free mode [29]. Other parameters: FDR < 1% of peptide and protein, MBR function selected, and the remaining parameters set as default. Protein abundance data were normalized and binary logarithmically transformed to achieve a normal distribution. Proteins with missing values > 50% were excluded from the following analysis. After group annotation, protein filtering, transformation and missing value replacement, the normal distribution-based differential protein abundances between groups were analysed with ANOVA, and two groups were analysed with a two-tailed Student’s t test. Orthogonal partial least-squares discriminant analysis (OPLS-DA) was applied with the ‘ropls’ function in R (v.4.1.0). Proteins were considered significantly changed if they had a *p*-value < 0.05. Gene Ontology (GO) analysis and Kyoto Encyclopedia of Genes and Genomes (KEGG) databases were conducted using the Database for Annotation, Visualization and Integrated Discovery (https://www.bioladder.cn/; http://www.bioinformatics.com.cn/) to identify enriched proteins. GO analysis was mainly divided into three categories: cellular component, biological process, and molecular function.

### Mitochondrial function measurement

The concentration of ATP was determined using an ATP assay kit (Nanjing Jiancheng Bioengineering Institute, China) according to the manufacturer’s instructions. The activity of mitochondrial complex I was determined by a mitochondrial respiratory chain (MRC) complex I activity assay kit (Beijing Solarbio Science & Technology Co., Ltd, China). Briefly, the respective reaction buffers were added to the mitochondria isolated from the mouse brain. The mixture was transferred to a prewarmed quartz cuvette and immediately put into a spectrophotometer. Then, the activity of mitochondrial complex I was determined by monitoring the decrease in nicotinamide adenine dinucleotide (NADH) at 340 nm for 2 min. The concentration of malondialdehyde (MDA) were measured using a lipid peroxidation MDA assay kit (Beyotime, Shanghai, China) according to the manufacturer’s instructions.

### Real-time quantitative PCR

Thalamus tissues were homogenized in TRIzol reagent (Invitrogen) and processed using Zymo Direct-zol RNA Microprep kit according to the manufacturer’s instructions [30]. RNA from each sample were then reverse-transcribed using SuperScript III First-Strand Synthesis System (Invitrogen). Quantitative gene expression data were acquired on Bio-rad real-time PCR system. The primers for each analyzed gene were listed: *Fundc1*, forward primer: ACATTGTGATATCCAGCGG CTTCG and reverse primer: TGCCACAGTCTTCCTCTCATTGTTG; *Fis1*, forward primer: CCTGGTTCGAAGCAAATACAAT and reverse primer: CTTTTCATATTCC TTGAGCCGG; *Mid49*, forward primer: GTGACGGCTGACCATATCCAACTC and reverse primer: CTCACGACGAACCAGGAAGAAACC; *OPA1*, forward primer: CTTACATGCAGAATCCTAACGC and reverse primer: CCAAGTCTGTAACA ATACTGCG; *PGC1α*, forward primer: GGATATACTTTACGCAGGTCGA and reverse primer: CGTCTGAGTTGGTATCTAGGTC; *TFAM*, forward primer: GTG AGCAAGTATAAAGAGCAGC and reverse primer: CTGAACGAGGTCTTTTTG GTTT; *CHOP*, forward primer: CTCCAGATTCCAGTCAGAGTTC and reverse primer: ACTCTGTTTCCGTTTCCTAGTT; *HSP10*, forward primer: GGCCCGA GTTCAGAGTCC and reverse primer: TGTCAAAGAGCGGAAGAAACTT.

### Transmission electron microscope (TEM)

Mice were anesthetized with sodium pentobarbital (50 mg/kg) and perfused with 0.1 M PBS solution (pH 7.4) followed by precooled 2.5% glutaraldehyde solution. The ventral posteromedial thalamic nucleus (VPM) was collected and cut into 1 × 1 × 1 mm tissue blocks. The tissue was fixed with 2.5% glutaraldehyde solution and then fixed in 10 g/L osmium tetroxide solution. The sample was dehydrated with a graded series of ethanol and embedded in Eponate 12 epoxy resin. Thin sections were counterstained with uranyl acetate and lead citrate. The micrographs were taken with TEM (HITACHI-HT7700, Japan).

### Statistical analysis

Data are presented as the mean ± SEM. All data were statistically analysed and graphed by GraphPad Prism (version 8; GraphPad Software, Inc., San Diego, CA). The Shapiro‒Wilk test was applied to analyse the data for normality. An independent-sample t test was performed to assess differences between two groups. Comparison among three or more groups were investigated by one-way ANOVA followed by Tukey’s multiple comparison test to detect pairwise between group differences. Time courses of nociceptive behavioral data were analysed by two-way ANOVA with the Bonferroni post hoc test. *p* < 0.05 was considered to be statistically significant.

## Results

### Repeated NTG stimulation led to CM-like phenomenology in mice

With repeated administration of NTG, mice exhibited a decreased trend with time in the basal mechanical and thermal thresholds (i.e., basal allodynia developed over time). Two hours after each single injection of NTG, an acute allodynia response was observed in mice. To evaluate the photophobic behavior of mice, a light-aversive test was applied. We found that mice in both groups spent significantly more time in the dark side of the box. Compared with those in the VEH group, mice in the NTG group showed a significant decrease in both the time spent in the light chamber and the number of transitions (Fig. 1). Together, these behavioral data indicated that a reliable mouse model of CM was established.

### Differential expression and quantitative analysis of proteins in five brain regions

**Fig. 3:**
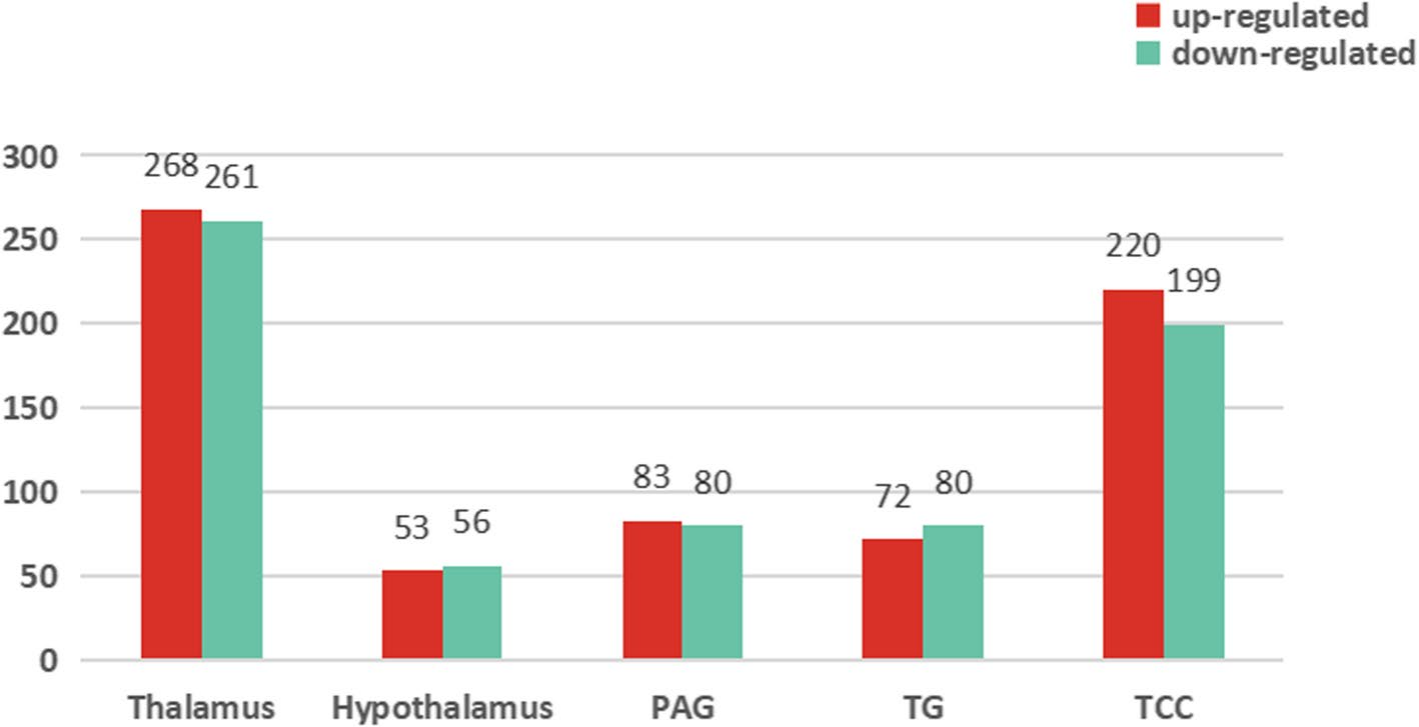
Quantitative analysis of DEPs between the NTG and VEH groups in five brain regions. The bar graphs showed the number of DEPs in the thalamus, hypothalamus, PAG, TG and TCC, respectively, in which red represented up-regulated proteins and green represented down-regulated proteins. The filtering criteria based on the expression fold change value > 1.5 or < 0.67 and the *p*-value < 0.05. (*n* = 6 per group)

Label-free quantitative proteomics was performed to reveal the NTG-induced DEPs in the brain. An explicit separation was shown in the thalamus, hypothalamus, PAG, TG and TCC between the NTG and VEH groups in the OPLS-DA model (Fig. 2). This figure shows volcano plots and clustering heatmaps of the DEPs in each brain region between the two groups. The number of proteins identified in each brain region of each sample is shown in Table 1, with a minimum of 6203 proteins and a maximum of 8225 proteins. Based on the filtering criteria of a fold change (FC) value > 1.5 or < 0.67 and a *p*-value < 0.05, 529, 109, 163, 152 and 419 DEPs between two groups were screened in five brain regions respectively. The number of DEPs in the thalamus was the highest. Compared with the VEH group, the NTG group had 268 up-regulated proteins and 261 down-regulated proteins (Fig. 3).

**Table 1:** Quantitative analysis of proteins identified in five brain regions

Group	Brain regions				
	Thalamus	Hypothalamus	PAG	TG	TCC
**VEH**	7564	7955	7459	6682	6940
	7698	7956	7473	7195	6853
	7724	8080	7766	6679	6928
	7759	8023	7704	6730	6945
	7854	8225	7507	6599	7087
	8058	8193	7758	6568	7148
**NTG**	7825	7919	7414	6407	6915
	7709	8159	7782	6743	7020
	7921	7610	7342	6909	6895
	7579	7922	7401	6203	6699
	7575	7737	7790	6639	7028
	7821	7945	7371	6655	6956

### Functional analysis of DEPs in different brain regions in the two groups

**Fig. 4:**
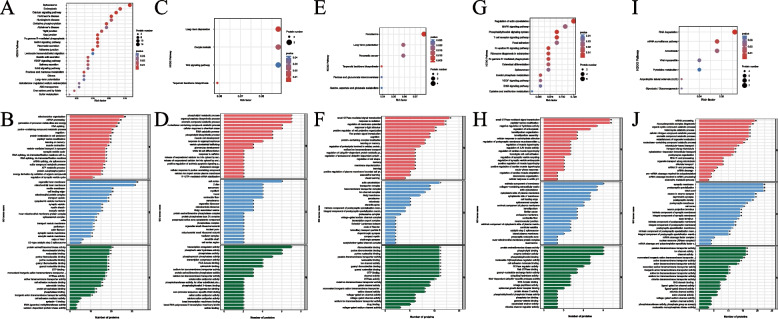
GO and KEGG pathway enrichment analysis of the DEPs between the NTG and VEH groups in five brain regions. KEGG analysis of the DEPs in the thalamus (**A**), hypothalamus (**C**), PAG (**E**), TG (**G**) and TCC (**I**). GO annotation of the DEPs in the thalamus (**B**), hypothalamus (**D**), PAG (**F**), TG (**H**) and TCC (**J**). (*n* = 6 per group)

We performed GO annotation and KEGG pathway analysis to elucidate the function of the DEPs (Fig. 4).

#### Thalamus

According to GO annotation, the enriched cellular components were mainly the mitochondrial inner membrane, mitochondrial protein complex, and inner mitochondrial membrane protein complex. The biological processes were mainly involved in mitochondrion organization, generation of precursor metabolites and energy, ATP metabolic process, and energy derivation by oxidation of organic compounds. The molecular functions included protein serine/threonine kinase activity, guanyl ribonucleotide binding, guanyl nucleotide binding, GTP binding, and GTPase activity. The top 25 significantly enriched pathways included oxidative phosphorylation, tight junctions, gap junctions, leukocyte transendothelial migration, adherens junctions, and long-term potentiation.

#### Hypothalamus

GO annotation showed that the enriched cellular components were the cell cortex, organellar ribosome, mitochondrial ribosome and Golgi-associated vesicle. The biological processes were enriched in phospholipid metabolic process, organophosphate biosynthetic process, phospholipid biosynthetic process and positive regulation of extrinsic apoptotic signaling process. The molecular functions included transcription coregulator activity, phosphoric ester hydrolase activity, and phosphatase activity. KEGG enrichment analysis showed that the DEPs were mainly enriched in long-term depression, the Wnt signaling pathway, and terpenoid backbone biosynthesis.

#### PAG

The enriched cellular components were transporter complex, transmembrane transporter complex, and ion channel complex. The biological processes were enriched in small GTPase mediated signal transduction, regulation of membrane potential, and response to light stimulus. The molecular functions included passive transmembrane transporter activity, ion channel activity, GTP binding, channel activity, and GTPase activity. KEGG enrichment analysis showed that the DEPs were mainly enriched in peroxisome, long-term potentiation, terpenoid backbone biosynthesis and alanine, aspartate and glutamate metabolism pathways.

#### TG

The cellular components were enriched in extrinsic component of membrane, collagen − containing extracellular matrix, and cytoplasmic side of plasma membrane. The biological processes were enriched in small GTPase mediated signal transduction, peptidyl − serine modification, and negative regulation of hydrolase activity. The molecular functions included protein serine/threonine kinase activity, phospholipid binding, and enzyme activator activity. KEGG enrichment analysis showed that the DEPs were mainly enriched in regulation of actin cytoskeleton, MAPK signaling pathway, and phosphatidylinositol signaling system.

#### TCC

The enriched cellular components were the synaptic membrane, postsynaptic specialization, distal axon, and neuron to neuron synapse. The biological processes were enriched in mRNA processing, ribonucleoprotein complex biogenesis, and organic cyclic compound catabolic process. The molecular functions included passive transmembrane transporter activity, ion channel activity, and monovalent inorganic cation transmembrane transporter. KEGG enrichment analysis showed that the DEPs were mainly enriched in RNA degradation, the mRNA surveillance pathway, and pyrimidine metabolism.

Overall, functional analysis of DEPs showed few signaling pathways shared by all five brain regions, suggesting that the pathogenesis underlying migraine may be different in different brain regions. The signaling pathway of mitochondrial oxidative phosphorylation was significantly changed only in the thalamus.

### Impaired energy metabolism in the thalamus in the NTG group

**Fig. 5:**
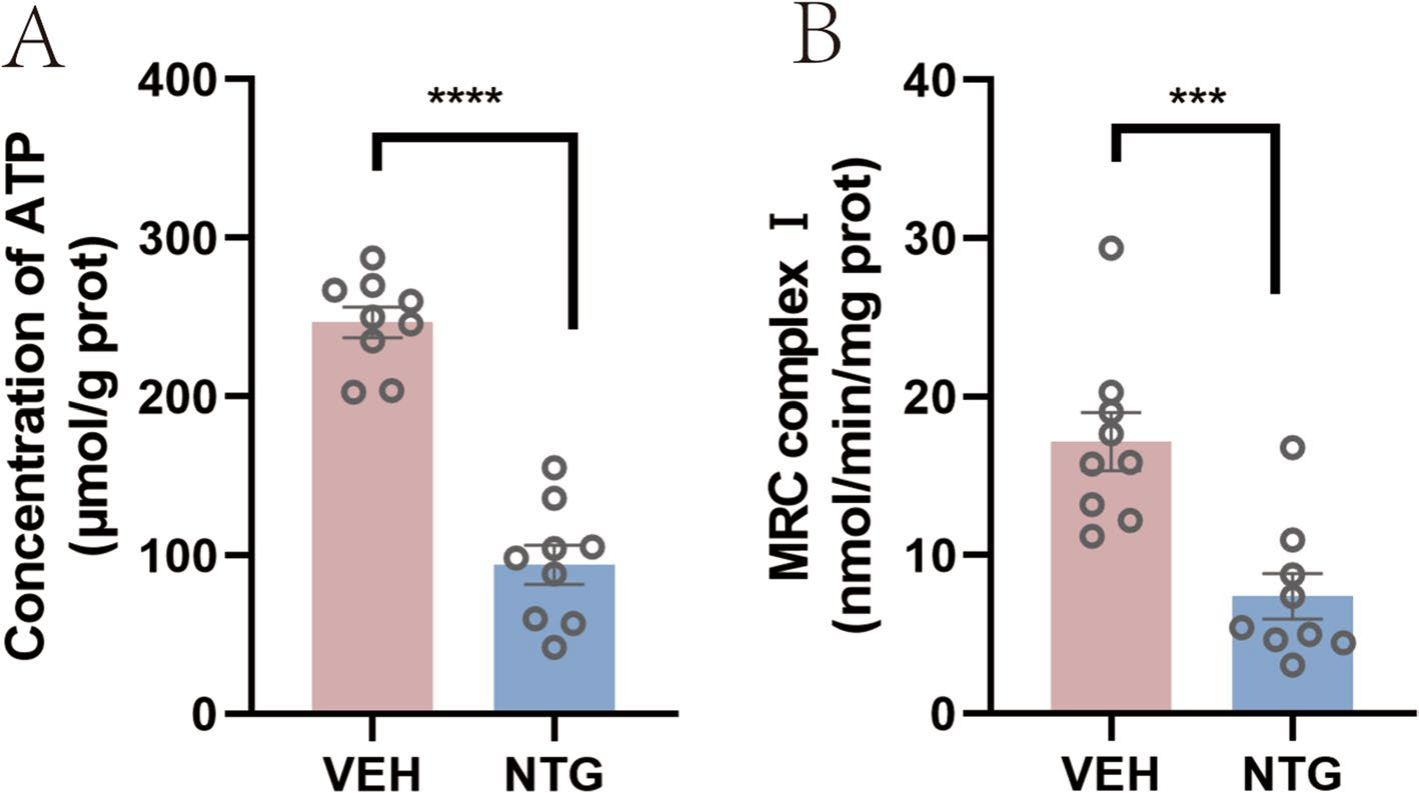
Impaired energy metabolism in the thalamus in the NTG group. The concentration of ATP (**A**) and the activity of MRC complex I (**B**) were measured in the thalamus in the NTG and VEH groups. The data are presented as the mean ± SEM. Independent-sample t test. (*n* = 9 per group, ***p < 0.001, ****p < 0.0001)

To clarify the difference in mitochondrial energy metabolism in the thalamus between the NTG and VEH groups, we measured the concentration of ATP and activity of MRC complex I in the thalamus of mice. The results showed that both the concentration of ATP and activity of MRC complex I in the NTG group were significantly lower than those in the VEH group (*p* < 0.0001 for ATP; *p* < 0.001 for MRC complex I) (Fig. 5).

### Morphological damage to mitochondria in the VPM of the thalamus in the NTG group

**Fig. 6:**
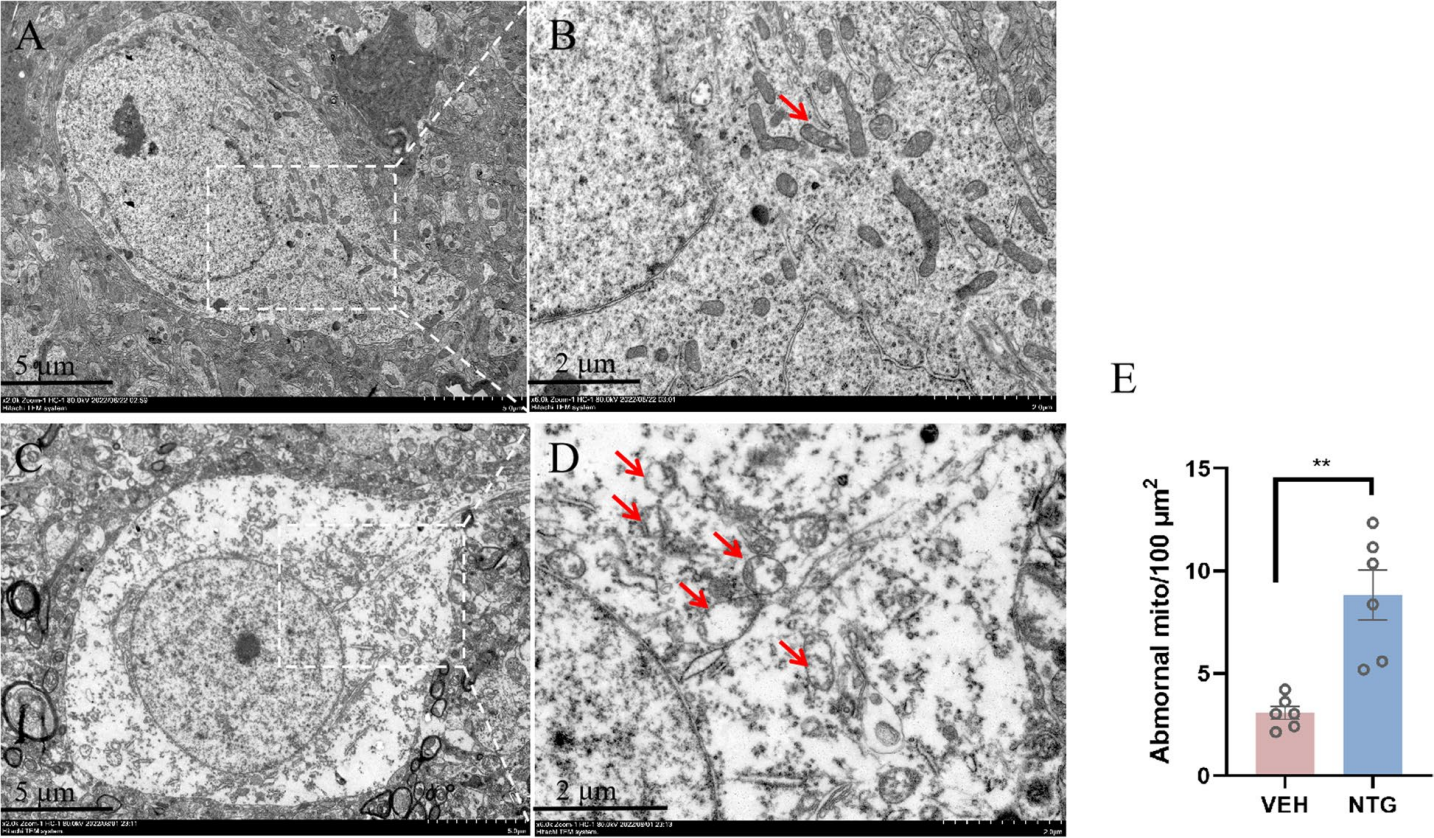
Comparison of mitochondrial morphology in VPM of the thalamus between the NTG and VEH groups. **A** and **B** Representative micrographs of mitochondria in VPM of the VEH group, Scale bar, 5 μm (**A**) and 2 μm (**B**). **C** and **D** Representative micrographs of mitochondria in VPM of the NTG group, Scale bar, 5 μm (**C**) and 2 μm (**D**). Abnormal mitochondria were indicated by red arrows. **E** The number of abnormal mitochondria per 100 µm^2^ is calculated in two groups. The data are presented as the mean ± SEM. Independent-sample t test. (*n* = 6 per group, **p < 0.01)

We performed TEM to observe the morphology of mitochondria within the VPM of the thalamus between the two groups. Compared to the VEH group, the NTG group showed a higher degree of mitochondrial damage, as evidenced by moderate or severe swelling, vacuolization, irregular morphology, and broken or disrupted cristae (reduced mitochondrial cristae). The number of abnormal mitochondria per 100 µm^2^ in the NTG group was significantly increased compared with that in the VEH group (*p* < 0.01) (Fig. 6).

### Disturbed mitochondrial quality control in the thalamus in the NTG group

**Fig. 7:**
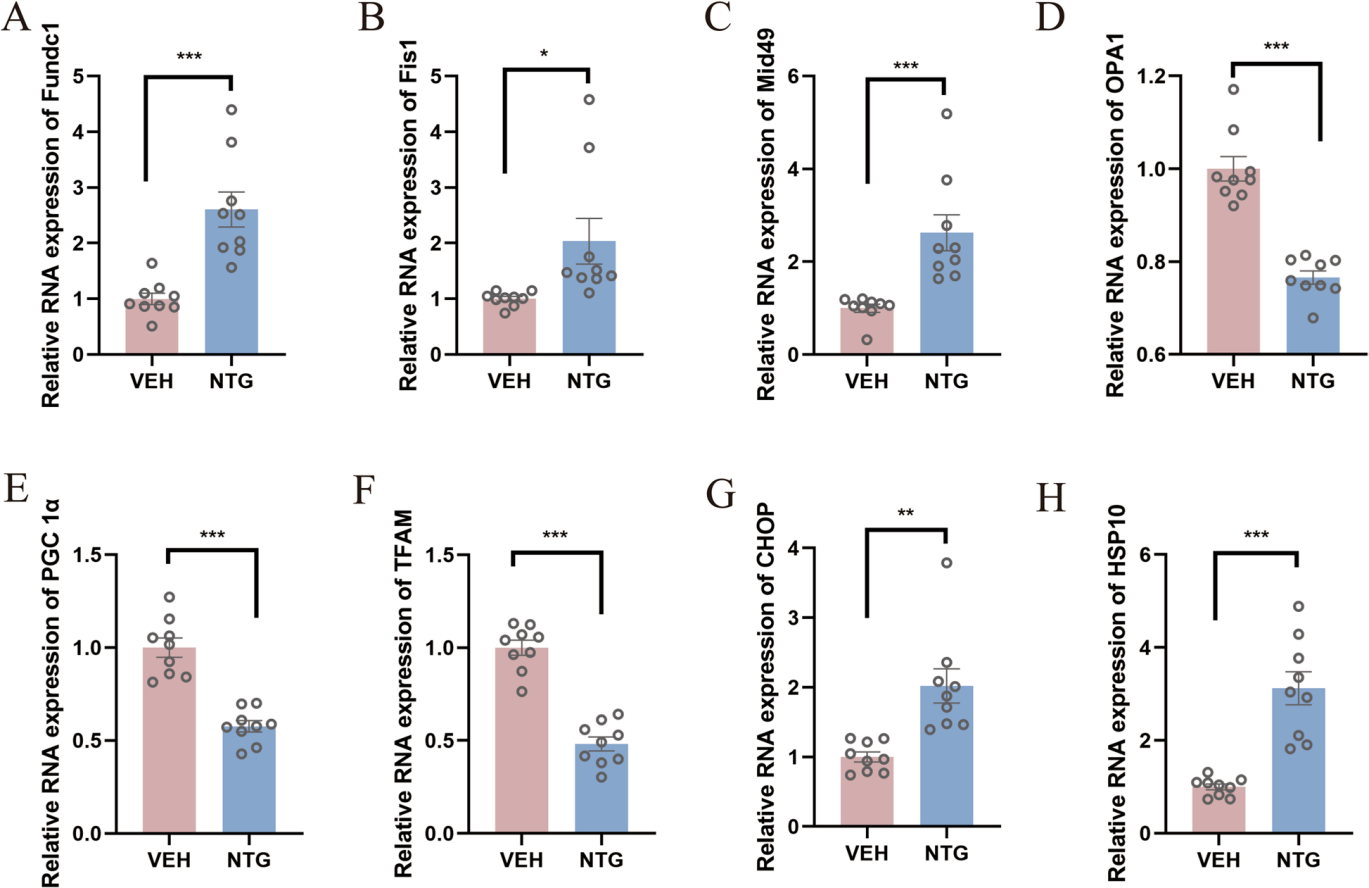
Disturbed mitochondrial quality control in the thalamus in the NTG group. The relative expression of *Fundc1* (**A**), *Fis1* (**B**), *Mid49* (**C**), *OPA1* (**D**) transcription was measured in the thalamus of mice. *PGC1α* (**E**), *TFAM* (**F**), *HSP10* (**G**) and *CHOP* (**H**) transcription levels were evaluated. The data are presented as the mean ± SEM. Independent-sample t test (*n* = 9 per group *p < 0.05, **p < 0.01, ***p < 0.001)

To determine whether mitochondrial quality control (MQC) is implicated in CM, molecular investigation was performed to evaluate mitophagy, fission, fusion, biogenesis and mitochondrial unfolded protein response (UPR). Our data illustrated both *Fis1* and *Mid49*, two major mitochondrial fission markers were upregulated and *OPA1*, *PGC1α* and *TFAM* respectively related to fusion and biogenesis, reduced in CM (*p* < 0.05, Fig. 7), implicating the balance among fission, fusion and biogenesis was disturbed. Interestingly, mitophagy (including *Fundc1*) and mitochondrial UPR (*CHOP* and *HSP10*) were strikingly elevated in response to repeated NTG injection (*p* < 0.05, Fig. 7).

### UA ameliorated damaged mitochondria and CM-like phenomenology induced by repeated NTG

**Fig. 8:**
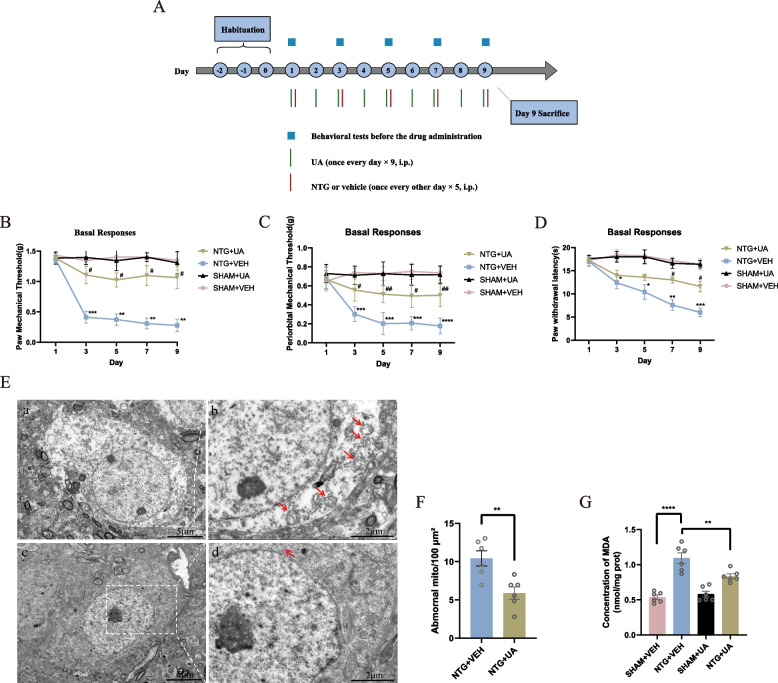
UA ameliorated allodynia in mice by reducing mitochondrial impairment and improving mitochondrial function. **A** Flow chart of the experiment. **B**-**D** The basal mechanical pain thresholds of the hind paw (**B**), periorbital area (**C**) and the thermal pain threshold of the hind paw (**D**) during the injection of UA and NTG. Two-way ANOVA with the Bonferroni post hoc test. *p < 0.05, **p < 0.01, ***p < 0.001, ****p < 0.0001 compared with the SHAM + VEH group; #*p* < 0.05, ##*p* < 0.01 compared with the NTG + VEH group. **E** Comparison of mitochondrial morphology in VPM of the thalamus between the NTG + VEH and NTG + UA groups. **a** and **b** Representative micrographs of mitochondria in VPM of the NTG + VEH group, Scale bar, 5 μm (**a**) and 2 μm (**b**). **c** and **d** Representative micrographs of mitochondria in VPM of the NTG + UA group, Scale bar, 5 μm (**c**) and 2 μm (**d**). Abnormal mitochondria were indicated by red arrows. **F** The number of abnormal mitochondria per 100 µm^2^ is calculated in two groups. Independent-sample t test. **p < 0.01. **G** After UA treatment, concentration of MDA induced by NTG administration decreased. One-way ANOVA and Tukey’s multiple comparison test. ****p < 0.0001: the NTG + VEH group vs. the SHAM + VEH group, **p < 0.01: the NTG + VEH group vs. the NTG + UA group. *n* = 6 per group. All data are presented as the mean ± SEM

To determine whether mitochondria could be regarded as key target, we compared the effects of UA intervention on allodynia induced by NTG. And then we observed that UA partially improved the basal mechanical and thermal pain thresholds induced by NTG (*p* < 0.05) and decreased damaged mitochondria (the concentration of MDA and the number of abnormal mitochondria per 100 µm^2^ decreased significantly in the NTG + UA group than NTG + VEH group), suggesting that the improvement of mitochondrial function has a certain therapeutic effect on allodynia in CM mice (Fig. 8).

## Discussion

Migraine is a highly prevalent and complex disorder. The underlying mechanism is unclear, but the trigeminovascular system is widely recognised as the pathophysiological basis of migraine attacks. The anatomy of the trigeminovascular pain pathway suggests that some important brain regions are activated, including the thalamus, hypothalamus, PAG, TG, and TCC [31]. In this study, we focused on the above five brain regions and revealed brain region-specific protein characteristics using proteomics analysis.

We found that compared to the VEH group, the NTG group showed significantly different brain protein profiles. The highest number of DEPs was found in the thalamus. Alterations in thalamic mitochondrial energy metabolism are particularly evident in brain region-specific protein profiles. In migraine pathogenesis, the thalamus is considered the central integration and processing center of nociceptive information [32]. The ventral posteromedial, posterior and lateral thalamic nucleus receive nociceptive inputs from the dura mater and relay the information to different cerebral cortices [33, 34]. Cutaneous allodynia in migraine is thought to be a consequence of sequential activation and sensitization of neurons along the trigeminovascular pathway. The sensitization of second-order neurons in the spinal cord mediates ipsilateral cephalic allodynia, and central sensitization of third-order neurons in the thalamus mediates contralateral and extracranial allodynia [35, 36]. In our study, the periorbital and hind paw pain thresholds of CM mice decreased, suggesting that repeated NTG stimulation could induce central sensitization of the thalamus. Photophobia is an important feature of migraine. The light-dark box test in this study showed significant light aversion in NTG-stimulated mice, supporting the validity of the CM mouse model constructed by intermittent administration of NTG. A study of neuronal projections of the retino-thalamo-cortical pathway showed that the photophobia is modulated at the level of the thalamus [33, 37].

Besides, mitochondria attracted our attention and we evaluated mitochondrial activity from three aspects: function, morphology, and quality control. TEM were used for observing mitochondrial morphology changes. The MRC complex I activity, ATP level and MDA content were used to evaluate mitochondrial function. The transcription levels of key genes were measured for MQC. These three aspects were mutually validating. Mitochondria abnormal morphology could affect the function and quality control, when mitochondrial function is regulated by morphology and quality control.

The concentration of ATP and the activity of complex I in the thalamus of the NTG group were significantly reduced compared with those of the VEH group. we observed different degree of mitochondrial damage in the thalamus of the NTG group, and most mitochondria showed moderate to severe damage through TEM. MQC is a delicate regulation system of mitochondria that could control mitochondrial quantity and quality. Repeated NTG injection could induce disturbed MQC, reflected in increased fission and mitophagy, abnormal UPR. Imbalanced MQC could affect the mitochondrial morphology and function, which are mirrored in abnormal TEM and enzyme activity. Although simple assays are used for evaluation, we think our data could suggest that mitochondrial dysfunction was linked with chronic migraine, which is supported by human plasma transcriptomic and metabolomic study [12] and other CM model [38].

However, the role of mitochondrial impairment, which is the cause or result of CM, is unclear. Therefore, we performed therapeutic interventions to elucidate the role of mitochondria in CM. UA is a natural compound produced by gut bacteria from ingested ellagitannins (ETs) and ellagic acid (EA), which is of great benefit to aging and chronic diseases [23]. UA enhances the homeostasis of mitochondria by increasing mitophagy and mitochondrial function, increasing antioxidative stress response and reducing detrimental inflammation [23, 39]. Based on previous research, UA can cross the blood-brain barrier and may be used for neurological diseases intervention. Recent studies have demonstrated that UA could alleviate neuropathic pain through improving mitophagy and exhibit a neuroprotective effect on neurodegenerative diseases [40–42]. This neuroprotective effect was observed in our CM model-After administrating UA into NTG-induced mice, we found UA could greatly ameliorate damaged mitochondria and CM-like phenomenology induced by repeated NTG administration. Taken together, these data indicated that mitochondria in the trigeminovascular system seems to play a key role in pathogenesis of CM.

Of course, our conclusion was supported by other groups’ studies. Based on a classic migraine rat model established by repeated dural infusions of inflammatory soup (IS), Xin Dong et al. [38] observed abnormal mitochondrial dynamics and damaged mitochondrial biogenesis in the TG. Based on NTG-induced CM model, Barbosa IR et al. [43, 44] reported that mitochondrial metabolism was influenced by NTG in the brain cortices and TG. However, fructose supplementation led to changes in brain metabolism, lessened the migraine-like painful symptoms, and reduced serum CGRP levels, which might imply that an increased energy supply could be somewhat favorable in migraine. Li R et al. [45] found that NTG caused mitochondrial energy metabolism dysfunction and mitochondrial biogenesis disruption in the spinal trigeminal nucleus in NTG-treated rats, which could be alleviated by valproate. Yazğan Y et al. [46] reported that NTG induced mitochondrial dysfunction in TG in the experimental NTG migraine mouse model.

Not only CM mouse models presented abnormal mitochondria took part in CM pathogenesis, but also human research provided the similar results. The latest research suggests that migraine is a brain dysfunction disorder, which occurs in genetically predisposed individuals with a mismatch between the brain’s energy reserve and workload [5, 47–49]. A series of ^31^P­-MRS studies consistently highlighted the impairment of mitochondrial oxidative phosphorylation in the brains of migraine patients as evidenced by increased ADP levels and decreased phosphorylation potential (an index for readily available free energy) [50–52]. Using a modified MRS method, Harmen et al. [17] found that ATP levels decreased significantly in the resting-state in patients with migraine without aura. Interestingly, the lowest ATP level was detected in those patients who had the highest attack frequency, suggesting modest associations between brain hypometabolism and migraine susceptibility. Ashina et al. [53] believe that modulation of nociceptive transmission by ATP-sensitive potassium (K_ATP_) channels may be a final common pathway in the genesis of a migraine attack. K_ATP_ channels are regulated by the intracellular ATP/ADP ratio, and the levels of cyclic adenosine monophosphate (cAMP) and cyclic guanosine monophosphate (cGMP), thereby supporting the hypothesis that brain mitochondrial energy metabolism is involved in the pathogenesis of migraine.

A ^1^ H-MRS study reported decreased concentrations of N-acetyl-aspartate (NAA) in the thalamus of patients with migraine without aura [54]. NAA is synthesized and located prevalently in neural mitochondria, and has been regarded as a marker of mitochondrial function [55, 56]. The decrease in NAA observed may indicate mitochondrial dysfunction in the thalamus, leading to abnormal energy metabolism in migraine. Eising et al. [57] integrated migraine genome-wide association study (GWAS) data through the International Headache Genetics Consortium [58] with high-resolution spatial gene expression data of normal adult brains from the Allen Human Brain Atlas [59] to identify specific brain regions and molecular pathways that are possibly involved in migraine pathophysiology. The results suggested that migraine-associated genes are involved in energy supply in the cortex and thalamus, which is consistent with the findings of our study.

The present study provides a comprehensive assessment of protein signatures across five key subcortical brain regions in CM mice, indicating an important role of thalamic mitochondrial function in migraine chronification. We speculate that migraine chronification may be due to a decline in thalamic energy reserves caused by impaired mitochondrial metabolism, thus increasing the susceptibility to attacks.

There are still some limitations in this study. Although the mouse model established by administration of NTG can partially mimic human migraine attacks, the current findings in mice cannot be extended to humans. Considering the effect of female hormones on pain processing in migraine, we performed the experiments using male mice. However, migraine is a female-prevalent disorder, so the results of this study did not take into account the role of female hormones. Despite the link between mitochondria and CM-related phenotypes, it has yet to be determined the molecular mechanism that the mitochondrial impairment could affect CM.

## Conclusions

Collectively, our study reveals that mitochondrial damage in the thalamus is involved in central sensitization of CM in mice. Further studies are needed to explore the specific molecular mechanism between mitochondrial metabolism and CM.

## Data Availability

The datasets generated and analyzed during the current study are available from the corresponding author on reasonable request.

## Abbreviations

NTG: Nitroglycerin
DEPs: Differentially expressed proteins
LC‒MS/MS: Liquid chromatography-tandem mass spectrometry
TEM: Transmission electron microscope
UA: Urolithin A
PAG: Periaqueductal grey
TG: Trigeminal ganglion
TCC: Trigeminocervical complex
GBD: Global Burden of Diseases
CM: Chronic migraine
EM: Episodic migraine
MELAS: Mitochondrial encephalopathy with lactic acidosis and stroke-like episodes
CSD: Cortical spreading depression
CSF: cerebrospinal fluid
i.p.: Intraperitoneal
FASP: Filer-aided sample preparation
TFA: Trifluoroacetic acid
OPLS-DA: Orthogonal partial least-squares discriminant analysis
GO: Gene ontology
KEGG: Kyoto encyclopedia of genes and genomes
MRC: Mitochondrial respiratory chain
NADH: Nicotinamide adenine dinucleotide
MDA: Malondialdehyde
VPM: Ventral posteromedial thalamic nucleus
FC: Fold change
MQC: Mitochondrial quality control
UPR: Mitochondrial unfolded protein response
ETs: Ellagitannins
EA: Ellagic acid
IS: Inflammatory soup
K_ATP_: ATP-sensitive potassium
cAMP: Cyclic adenosine monophosphate
cGMP: Cyclic guanosine monophosphate
NAA: N-acetyl-aspartate
GWAS: Genome-wide association studies
